# Mechanisms Controlling the Effects of Bevacizumab (Avastin) on the Inhibition of Early but Not Late Formed Corneal Neovascularization

**DOI:** 10.1371/journal.pone.0094205

**Published:** 2014-04-08

**Authors:** Wei-Li Chen, Yan-Ming Chen, Hsiao-Sang Chu, Chung-Tien Lin, Lu-Ping Chow, Chih-Ta Chen, Fung-Rong Hu

**Affiliations:** 1 Department of Ophthalmology, National Taiwan University Hospital, Taipei, Taiwan; 2 Center of Corneal Tissue Engineering and Stem Cell Biology, National Taiwan University Hospital, Taipei, Taiwan; 3 Department of Ophthalmology, E-Da Hospital, I-Shou University, Kaohsiung, Taiwan; 4 Department of Veterinary Medicine, College of Bio-Resource and Agriculture, National Taiwan University, Taipei, Taiwan; 5 Institute of Biochemistry and Molecular Biology, College of Medicine, National Taiwan University, Taipei, Taiwan; Wayne State University, United States of America

## Abstract

**Purpose:**

To evaluate the effects and underlying mechanisms of early and late subconjunctival injection of bevacizumab on the inhibition of corneal neovascularization (NV).

**Methods:**

Corneal NV was induced by closed eye contact lens wear followed by a silk suture tarsorrhaphy in rabbits. Weekly subconjunctival injections of bevacizumab (5.0 mg) for 1 month were started immediately (early treatment group) or 1 month after induction of corneal NV with continuous induction (late treatment group). The severity of corneal NV was evaluated. Immunostaining was used to evaluate the intracorneal diffusion of bevacizumab, and the existence of pericytes and smooth muscle cells around the NV. The expression of AM-3K, an anti-macrophage antibody, vascular endothelial growth factor (VEGF) with its receptors (VEGFR1 and VEGFR2), and vascular endothelial apoptosis were also evaluated. Western blot analysis was performed to quantify the expression level of VEGF, VEGFR1 and VEGFR2 on corneal epithelium and stroma in different groups.

**Results:**

Early treatment with bevacizumab inhibited corneal NV more significantly than late treatment. Intracorneal diffusion of bevacizumab was not different among different groups. Immunostaining showed pericytes and smooth muscle cells around newly formed vessels as early as 2 weeks after induction. Immunostaining and Western blot analysis showed that VEGF, VEGFR1, and VEGFR2 on corneal stroma increased significantly in no treatment groups and late treatment groups, but not in early treatment group. Bevacizumab significantly inhibited macrophage infiltration in the early but not late treatment group. Sporadic vascular endothelial apoptosis was found at 4 weeks in the late but not early treatment group.

**Conclusions:**

Early but not late injection of bevacizumab inhibited corneal NV. Late injection of bevacizumab did not alter macrophage infiltration, and can't inhibit the expression of VEGF, VEGFR1, and VEGFR2 on corneal vessels. The inhibition of corneal NV in early treatment group does not occur via vascular endothelial apoptosis.

## Introduction

Normal cornea is avascular. However, corneal neovascularization (NV) can occur as a consequence of anterior segment inflammation, injury, and ischemia. This unwanted pathological response can cause visual impairment [1]–[5] or other conditions such as corneal edema, corneal scarring, lipid deposition, increased risk of graft rejection after corneal transplantation, and bleeding during corneal flap preparation in laser in situ keratomileusis (LASIK) surgery. [1], [6]–[10]

Vascular endothelial growth factor (VEGF) induces corneal NV under pathological scenarios. [2], [5], [11]–[14] Numerous studies have shown that VEGF is a critical mediator of retinal and iris NV following injury and ischemia, and in diabetic retinopathy too. [15], [16] Increased VEGF mRNA levels in the epithelium were also observed in a rabbit model of closed eye contact lens (CL)-induced corneal NV. [17] Corneal epithelial and endothelial cells, vascular endothelial cells of limbal vessels, and fibroblasts and macrophages in scar tissue all excrete VEGF, especially in inflamed and vascularized corneas. [11], [14] The receptors of VEGF (VEGFR1 and VEGFR2) were also found in newly proliferating vascular endothelial cells in inflamed cornea. [18]–[21]

Several studies have shown that anti-VEGF agents can inhibit corneal NV. [22]–[29] One such inhibitor is bevacizumab, a humanized murine monoclonal antibody against all VEGF isoforms. [23], [30] Bevacizumab has been used to treat metastatic colorectal cancer, [31] diabetic retinopathy, [32]–[34] choroidal NV in pathologic myopia, [35] exudative age-related macular degeneration (ARMD), [36]–[38] and corneal NV in some circumstances. [39]–[41] Recently, we reported that subconjunctival injection of bevacizumab inhibits the formation of corneal NV in various rabbit corneal NV models [23], [27] and showed that bevacizumab can be used to effectively treat lipid keratopathy in certain patients. [29] Furthermore, in rabbit corneas, we found that administration of bevacizumab injection immediately after limbal injury has better inhibitory effects on corneal NV than late treatment. [27] Papathanassiou et al. also found that subconjunctival administration of bevacizumab effectively inhibits corneal neovascularization in an experimental rabbit model, especially if administered early. [22] In spite of abundant studies demonstrating the inhibition of NV formation on cornea and other tissues, the effects of bevacizumab on the expression of VEGF and its receptors in the cornea have seldom been reported. [42]–[44]

Newly formed corneal vessels undergo maturation, which involves coverage of vascular corneal endothelial cells by pericytes and smooth muscle cells. Cursiefen et al. reported that 80% coverage by pericytes is achieved in 2 weeks. [45], [46] Furthermore, Gee et al. showed that pericyte coverage of mature vessels markedly influences tumor vessel response to anti-vascular therapy in a mouse model. [47] However, the effect of those cells on intracorneal diffusion and therapeutic effects of bevacizumab is not completely understood. In this study, we evaluated the influence of the initiation time of bevacizumab treatment on corneal NV inhibition. Digital photography was used to record changes in corneal NV. Immunohistochemistry was performed to evaluate intracorneal bevacizumab diffusion and the expression of VEGF, VEGFR1, VEGFR2, AM-3K (an anti-macrophage antibody), and vascular endothelial apoptosis. A better understanding of the effects and underlying mechanisms of early and late subconjunctival injection of bevacizumab may help establishing guidelines for bevacizumab use in corneal NV treatment.

## Materials and Methods

### Chemicals and antibodies

Bevacizumab (Avastin; 100 mg/4 mL) was purchased from Roche Pharmaceuticals (Welwyn Garden City, UK). For immunohistochemistry, donkey anti-human IgG–Cy3 antibody was used for the detection of bevacizumab (Jackson ImmunoResearch, West Grove, PA). Goat polyclonal antibody for CD31, Texas Red anti-goat secondary antibody, and fluorescein isothiocyanate (FITC) anti-mouse secondary antibody were purchased from Santa Cruz Biotechnology, Inc. (Santa Cruz, CA). Mouse monoclonal antibody for VEGF165 was purchased from Abcam (Cambridge, UK). The α-smooth muscle actin (αSMA) antibody, which recognizes perivascular smooth muscle cells and pericytes, [45], [48]–[51] was purchased from Abcam (Cambridge, UK). Goat polyclonal antibody for VEGFR1, goat polyclonal antibody for VEGFR2, and mouse monoclonal antibody for high-molecular-weight melanoma-associated antigen (HMWMAA), which is a specific marker for pericyte, [52] were purchased from R&D (Minneapolis, MN). Mouse monoclonal anti-human AM-3K antibody, an anti-macrophage antibody, [53], [54] was purchased from Cosmo Bio Co. Ltd. (Tokyo, Japan). For Western blot analysis, mouse-anti-human monoclonal antibody for VEGF was purchased from Abcam (Cambridge, UK), mouse monoclonal antibody for VEGFR1 was purchase from Chemicon (Temecula, CA), mouse-anti- human monoclonal antibody for VEFGR2 was purchased from Santa Cruz Biotechnology, Inc. (Santa Cruz, CA). Terminal deoxynucleotidyl transferase dUTP nick end labeling (TUNEL) staining kit was purchased from Biovision (Milpitas, CA).

### Animals

New Zealand albino rabbits (weight, 3.0–3.5 kg; age, 6 months) were used in this study. Use, care, and treatment of all animals followed the regulations of the ARVO Statement for the Use of Animals in Ophthalmic and Vision Research. The protocol was approved by the Committee for Animal Research of the National Taiwan University Hospital (Permit Number: 11-0329). All surgical procedures were performed with animals under general anesthesia induced by intramuscular injection of ketamine hydrochloride (35 mg/kg) and xylazine hydrochloride (5 mg/kg). The eyes were topically anesthetized with 0.5% proparacaine hydrochloride (Alcaine; Alcon, Fort Worth, TX) before manipulation. The right eye of each animal was used for the experiments and controls, and the left eye was left untreated. Twelve eyes (6 experimental and 6 control eyes) were included in each group. The corneas were inspected under a surgical microscope, and observations were recorded weekly after induction of corneal NV until the completion of the experiments.

### Closed eye contact lens (CL)-induced corneal NV

We used the closed eye CL method described by Mastyugin et al. [17] as a model of stable and extensive corneal NV. First, we placed hard CLs made of polymethylmethacrylate (PMMA; Boston; 7.6-mm base curve, 13-mm diameter) onto the right eye. To keep the lenses in place, 5–7 interrupted 5-0 silk sutures (Ethicon; Somerville, NJ) were placed through the superficial tarsus of both eyelids, with due care to avoid penetration or damage of the tarsal conjunctiva. The tarsorrhaphy closed approximately 80–90% of eyelid fissures. Corneal NV was photographed weekly after temporary removal of tarsorrhaphy and CLs. An operative microscope (OPMI Pico I; Carl Zeiss Meditec, Jena, Germany) was used to record the severity of corneal NV, and data were analyzed as described below. After the observation, CLs were replaced with re-suturing of tarsorrhaphy.

### Subconjunctival injection of bevacizumab

Subconjunctival injection of bevacizumab was administered under general anesthesia. The rabbits were divided into 4 groups: normal cornea (no CL wear or bevacizumab treatment); no treatment (closed eye CL wear but no bevacizumab treatment); early treatment (weekly bevacizumab administration for 1-month initiated immediately after closed eye CL wear at a dose of 5.0 mg [2.5 mg/0.1 mL each into the lower and upper subconjunctiva]); and late treatment (weekly bevacizumab administration for 1-month initiated 1 month after closed eye CL wear at a dose of 5.0 mg [2.5 mg each into the lower and upper subconjunctiva]. Closed eye CL induction of corneal NV continued while the subconjunctival injections were used.). In early and late treatment groups, an equal volume of saline solution (instead of bevacizumab) was injected in the control eyes. All experiments were repeated 6 times to ensure consistent results.

### Intracorneal diffusion of bevacizumab

To detect intracorneal diffusion of bevacizumab after subconjunctival injection, 5.0 mg of bevacizumab were injected into the lower and upper subconjunctiva (2.5 mg each). At day 1, 3, and 7 after injection, the eyes were enucleated for immunohistochemical studies. All experiments were repeated 3 times to ensure consistent results.

### Record of corneal neovascularization

Corneal NV was photographed weekly under an operative microscope (OPMI Pico I) after closed eye CL application. Photographs were graded by 2 blinded ophthalmologists for the extent of corneal NV. The percentage of involved corneal surface (PICS) of NV was quantified by image analysis software (Image J 1.40v; Wayne Rasband, Research Services Branch, National Institute of Mental Health, Bethesda, MD). [23], [27], [29] The area of NV was measured in terms of pixels, and its ratio to the entire corneal area was determined as the percentage of corneal NV. Centricity was defined as the length (mm) of the new vessel extending from the limbus toward the visual axis. The extent of corneal NV was defined as the number of clock-hours of limbus affected by NV (scores 1–12; score 1 = 1 clock-hour, score 2 = 2 clock-hours, and so on).

### Immunohistochemistry

Rabbit eyeballs were cryopreserved in optimal cutting temperature (OCT) embedding medium, and 8-µm cryosections were obtained for immunohistochemistry staining. Sections were air-dried at room temperature for 30 min and then fixed in 4% paraformaldehyde for 10 min. Sections were permeabilized with 0.4% Triton X-100 for 10 min and blocked by 1% bovine serum albumin with 4% fetal bovine serum (FBS) at 37°C for 2 h. To detect bevacizumab, donkey anti-human IgG–Cy3 antibody was added onto tissues and incubated overnight at 4°C. This polyclonal antibody, which binds to the heavy and light chains of humanized IgG, has been used to detect the diffusion of bevacizumab after injection. [23] The nuclei were double-stained with 4′,6-diamidino-2-phenylindole (DAPI). The normal cornea without NV induction but with subconjunctival injection of bevacizumab was used as the control. Perivascular and smooth muscle cells were identified using pericyte marker HMW-MAA [52] and αSMA antibodies. [45], [48]–[51] Goat polyclonal anti-CD31 antibody was used to detect vascular endothelial cells. [55]–[57] VEGF, VEGFR1, and VEGFR2 antibodies were used along with anti-CD31 antibody to evaluate the expression of the formers and their association with vascular endothelial cells. The infiltration of macrophages, as visualized using AM-3K, was also evaluated for its association with vascular endothelial cells. [53], [54]. TUNEL staining was used concomitantly with anti-CD31 antibody to evaluate the apoptosis of vascular endothelial cells. [58] The staining pattern of the tissue sections was observed by conventional fluorescence microscopy by using an Eclipse E800 Nikon Microscope with a VFM Epi-Fluorescence Attachment (Nikon, Melville, NY) equipped with a Spot Digital Camera and Spot version 1.1 CE software (Diagnostic Instruments, Sterling Heights, MI) or performed using a Zeiss LSM 510 Meta (Carl Zeiss, Heidelberg, Germany) confocal laser scanning microscopy. The images obtained by confocal microscopy were processed and assembled using Zeiss LSM Image Browser version 4.2 (Carl Zeiss). All experiments were repeated 3 times to ensure consistent results. To quantify the severity of AM-3K positive and apoptotic cells in each experimental condition, at least 6 fields in each experiment were examined by one single examiner and the results were evaluated for statistical significance with a Student's t-test (unpaired, two-tail). The results are expressed as the mean±standard error of the mean (SEM).A level of p<0.05 was considered as statistically significant.

### Western blot analysis

Western blot analysis was used to quantify the expression level of VEGF, VEGFR1 and VEGR2 in corneal epithelium and stroma. After sacrificing rabbits and obtaining the corneas, the corneal endothelium and Descemet's membrane was removed from the stroma. The corneal epithelium was collected by scrapping with a surgical razor blade. The corneal epithelial cells and the stroma tissue were stored at −80°C until protein extraction. During protein extraction, the corneal epithelial and stromal tissues were frozen in liquid nitrogen and grounded by disposable pestles in RIPA buffer (Thermo, Rockford, USA) with protease inhibitor (Roche Applied Science, Mannheim, Germany). After sonication, tissue lysates were centrifuged and the supernatant was collected, Ten micrograms of tissue lysates were equally loaded in each lane of an SDS-PAGE gradient gel in reducing conditions. Proteins were then transferred to nitrocellulose membranes (GE Healthcare, Buckinghamshire, UK). The membranes were blocked with 5% nonfat milk in 0.1%TBS-Tween solution, and then incubated with primary antibodies at 4 °C overnight, then horseradish peroxidase-conjugated secondary antibodies (Invitrogen, Carlsbad, USA). The blots were then developed using a chemiluminescence assay to visualize bound antibody (ECL; Millipore, Billerica, MA). The western blot analyses were quantified with NIH Image J. The blots were digitized with a flatbed scanner, and the band density was measured by using Image J. To account for loading variability, GADHP was used to normalize each sample. At least 4 independent experiments were performed and the results were evaluated for statistical significance with a Student's t-test (unpaired, two-tail). The results are expressed as the mean±standard error of the mean (SEM). A p value of <0.05 was considered statistically significant.

## Results

### Early treatment with bevacizumab can inhibit corneal NV

Corneal NV began to emerge from the rabbit limbus 1 week after closed eye CL application, and considerable corneal NV was observed approximately 2 weeks after closed eye CL application. PICS, centricity, and extent of corneal NV peaked at 5 to 7 weeks. Corneal NV did not progress with continuous CL application after week 7, but rather began to regress gradually or remained stationary thereafter (Table 1). In early treatment group, in which subconjunctival injection of bevacizumab was administered weekly from day 0 until 1 month after closed eye CL wear, a significant decrease in corneal NV compared to the control group was observed from week 3 to 4 after treatment (PICS, centricity, and extent of corneal NV: all *p*<0.01) (Table 1). No adverse ocular complications related to subconjunctival injection were observed.

**Table 1:** The effects of bevacizumab injections on the inhibition of corneal NV in different treatment groups.

Early Treatment Group						
Weeks after NV induction		0	1	2	3	4
Weeks after initial treatment		0	1	2	3	4
PICS	Control	0±0	15.4±3.2	31.1±6.2	51.5±3.1	74.8±6.9
	Treatment	0±0	5.3±0.9	6.9±0.6	11.2±2.2	13.6±2.9
	p-value	NS	<0.001	<0.01	< 0.0001	< 0.0001
Centricity	Control	0±0	1.6±0.4	2.0±0.3	3.8±0.5	5.2±0.2
	Treatment	0±0	1.2±0.3	1.2±0.4	1.5±0.4	1.7±0.5
	p-value	NS	NS	<0.05	<0.01	<0.01
Extent	Control	0±0	5.0±1.0	7.0±1.0	10.7±0.6	11.0±1.0
	Treatment	0±0	4.3±1.5	4.7±1.5	5.0±1.7	5.3±1.2
	p-value	NS	NS	NS	<0.01	<0.01

Late Treatment Group						
Weeks after NV induction		4	5	6	7	8
Weeks after initial treatment		0	1	2	3	4
PICS	Control	72.2±4.6	84.0±5.0	84.9±5.0	84.4±4.9	72.4±4.9
	Treatment	69.2±5.5	73.7±5.9	75.1±6.0	72.7±6.5	67.7±6.5
	p-value	NS	NS	NS	NS	NS
Centricity	Control	5.2±0.2	5.3±0.2	5.3±0.2	5.2±0.2	5.0±0.2
	Treatment	4.9±0.1	5.0±0.1	5.0±0.1	5.0±0.1	4.9±0.1
	p-value	NS	NS	NS	NS	NS
Extent	Control	11.0±1.0	11.3±1.2	11.7±0.6	12.0±0	12.0±0
	Treatment	10.7±0.6	11.3±0.6	11.7±0.6	11.7±0.6	11.7±0.6
	p-value	NS	NS	NS	NS	NS

NV: Neovascularization. PICS: Percentage of involved corneal surface area. NS: Non-significant

### Late treatment with bevacizumab does not have an anti-angiogenic effect on actively induced corneal NV

In the late treatment group, in which subconjunctival injection of bevacizumab was administrated weekly from week 5 to 8 after induction of corneal NV, closed eye CL application was continued throughout the entire course of treatment. We found no significant decrease in corneal NV compared to the control group during the entire course of treatment (PICS, centricity, and extent of corneal NV: all *p*>0.05) (Table 1). No adverse ocular complications related to the subconjunctival injection were observed.

### Immunohistochemistry

**Figure 1:**
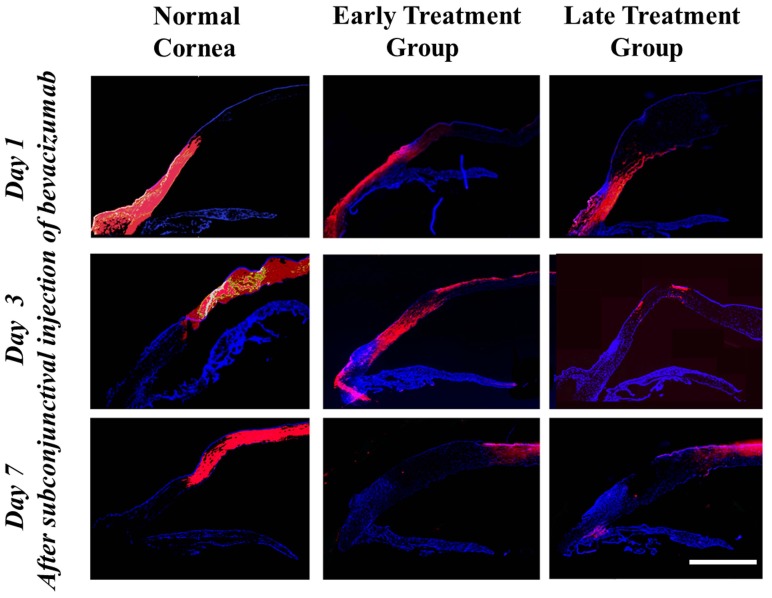
Immunohistochemical staining of bevacizumab after subconjunctival injection as detected by a Cy3-conjugated IgG antibody. Time-dependent diffusion of bevacizumab into the corneal stroma was observed. Normal: eyes without induction of corneal NV. Bevacizumab molecule staining was observed in the entire thickness of corneal stroma adjacent to the injection site in all groups 1 day after the injection. The distribution of bevacizumab staining moved to the more central cornea on day 3 in all groups. On day 7, bevacizumab staining decreased dramatically in the peripheral cornea but was detected in the central cornea in all groups. Purple red: bevacizumab molecule staining; blue: DAPI staining of cell nuclei. Scale bar: 4 mm.

Bevacizumab staining was detected in the entire corneal stromal thickness adjacent to the injection site in normal and early- and late-treated corneas at 1 day after administration of subconjunctival bevacizumab injection. Bevacizumab was distributed from the injection area to the central part of the cornea on day 3 after injection in all corneas. On week 1 after injection, bevacizumab staining was restricted to the central cornea and showed decreased density in all corneas (Figure 1). No specific staining was found in the negative control cornea in which subconjunctival injection of normal saline was administered instead of bevacizumab. This showed that the anti-IgG antibody specifically recognized the injected bevacizumab molecule.

**Figure 2:**
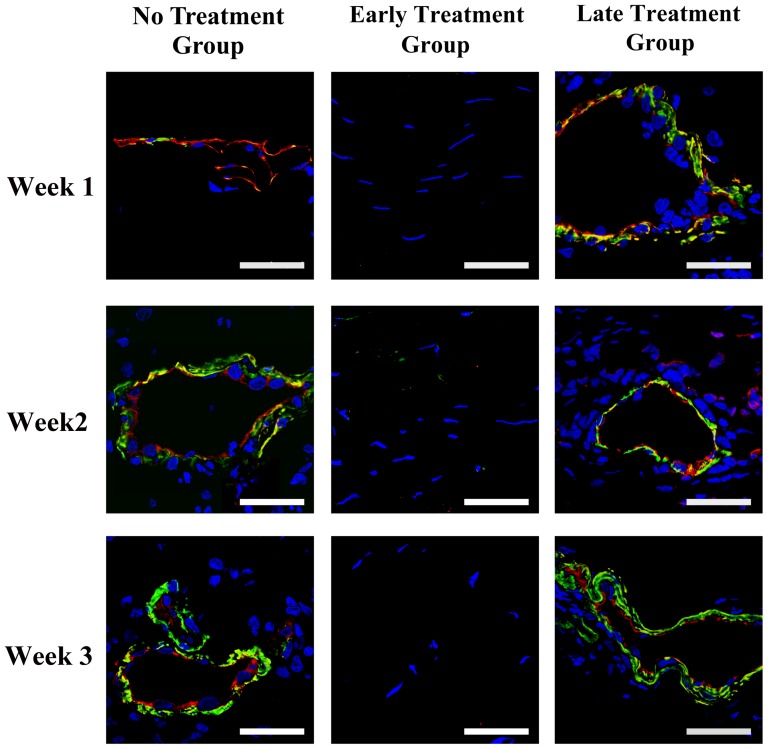
Immunostaining of CD31 andα-smooth muscle actin (αSMA) in vascularized rabbit corneas. Photos taken at 1, 2, and 4 weeks after the induction of corneal NV in no treatment group and 1, 2, and 4 weeks after bevacizumab injection in early and late treatment groups. Staining of CD31 surrounded by αSMA staining was found at 2 and 4 weeks in no treatment group, and week 1, 2, 4 after bevacizumab injection in late treatment group, which demonstrated the presence of smooth muscle cells around the vascular endothelial cells. Scale bar: 40 μm. Red: CD31; Green: αSMA; Blue: Hoechst33258 which stains nucleus.

**Figure 3:**
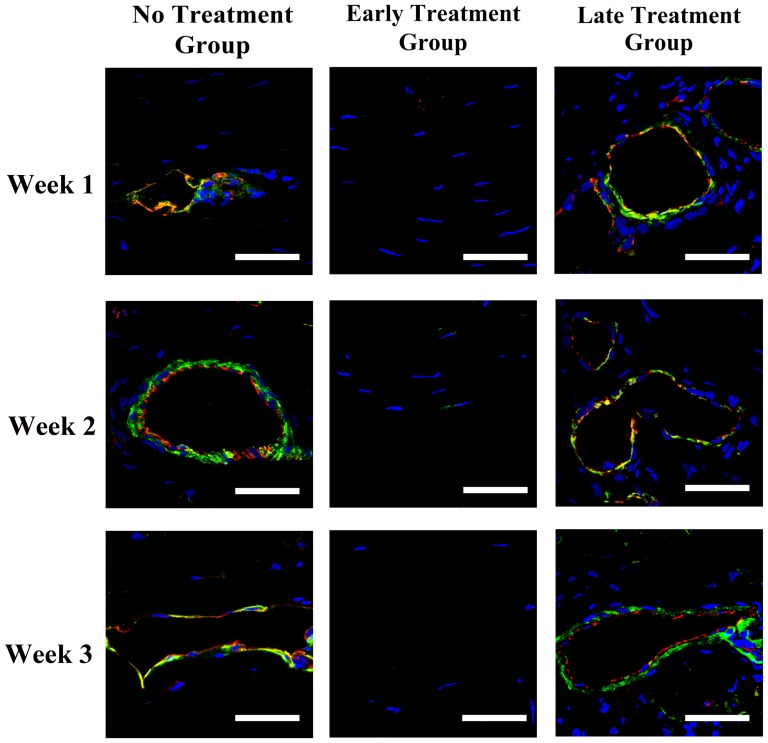
Immunostaining of CD31 and high molecular weight melanoma associated antigen (HMW-MAA) in vascularized rabbit corneas. Photos taken at 1, 2, and 4 weeks after the induction of corneal NV in no treatment group and 1, 2, and 4 weeks after bevacizumab injection in early and late treatment groups. Staining of CD 31 surrounded by HMW-MAA was found at week 2 and 4 in no treatment group, and week 1, 2, 4 after bevacizumab injection in late treatment group, which demonstrated the presence of pericytes around the vascular endothelial cells. Scale bar: 40 μm. Red: CD31; Green:HMW-MAA; Blue: Hoechst33258 which stains nucleus.

**Figure 4:**
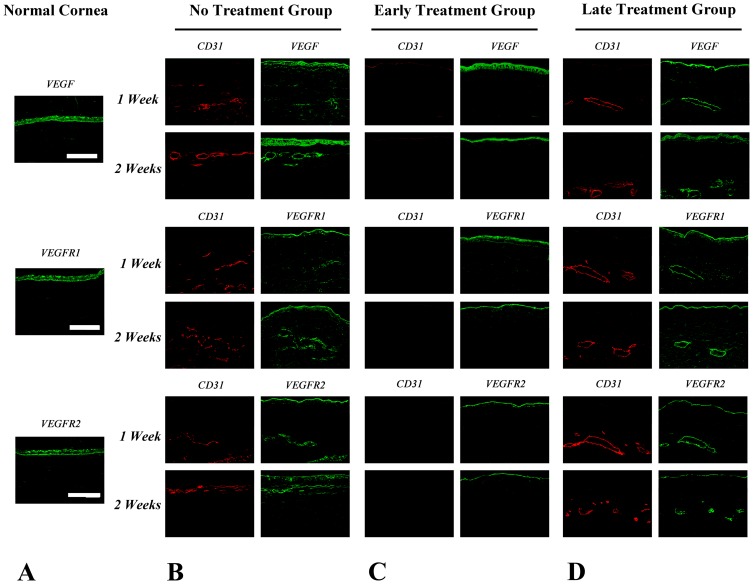
Immunohistochemistry of VEGF, VEGFR1 and VEGFR2 expression on cornea of all groups. (A) On normal cornea, VEGF, VEGFR1 and VEGFR2 were found to be presented on corneal epithelium. (B) In no treatment group, VEGF, VEGFR1 and VEGFR2 staining were found on corneal epithelium and corneal NV with co-localization with CD31 on week 1 and week 2. (C) In early treatment group, VEGF, VEGFR1 and VEGFR2 stained only on corneal epithelium on week 1 and week 2 after bevacizumab injection. No staining of VEGF, VEGFR1 and VEGFR2 on corneal vessels was found. (D) In late treatment group, staining of VEGF, VEGFR1 and VEGFR2 was found on corneal epithelium with co-localization with CD31 at 1 and 2 weeks after bevacizumab injection. Red: CD31; Green: VEGF, VEGFR1 and VEGFR2; Blue: Hoechst33258 which stains nucleus. Scale bar: 200 μm. Three repeats were performed to ensure consistent results. In early and late treatment groups: Week 1, 2 = Week 1, 2 after bevacizumab injection.

Immunohistochemistry showed both αSMA and HMW-MAA expression around CD31-positive vascular endothelial cells at week 2 and 4 after the induction of corneal NV in the no treatment group, and at week 1, 2, and 4 after bevacizumab injection in the late treatment group (Figure 2 and 3). This finding implied the presence of smooth muscle cells and pericytes around the vascular endothelial cells on the vessel walls of corneal NV as early as 2 week after closed eye CL wear. As for the molecules involved in VEGF signaling, the expression of VEGF, VEGFR1, and VEGFR2 was found strongly on corneal epithelium in normal, no treatment, and early- and late-treated corneas on week 1 and 2 (Figure 4). Co-staining of VEGF, VEGFR1, VEGFR2 and CD31, which implying vascular endothelial cells expressed VEGF, VEGFR1 and VEGFR2 was found on week 1 and week 2 in the no treatment and late treatment groups (Figure 4). However, there is no detectable staining of CD31, VEGF, VEGFR1, or VEGFR2 on cornel vascular endothelial cells in the early treatment group during the whole observational period (Figure 4).

**Figure 5:**
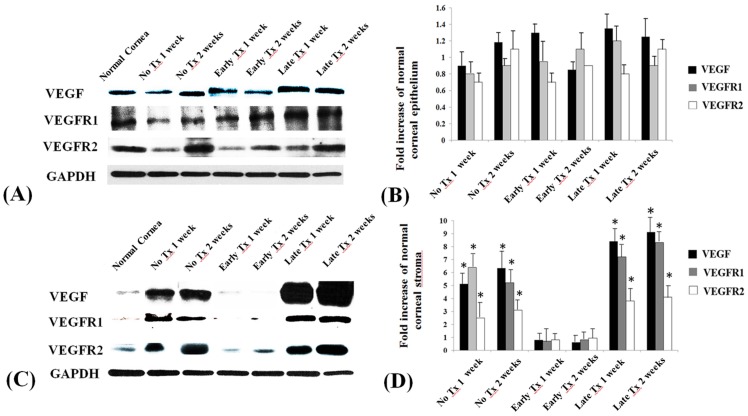
Quantification of VEGF, VEGFR1 and VEGFR2 expression levels on corneal epithelium and corneal stroma by Western blot analysis. (A) The Western blot analysis of VEGF/VEGFR1/VEGFR2 on corneal epithelium. (B) Densitometry showing the fold increase of VEGF/VEGFR1/VEGFR2 expression levels compared to normal corneal epithelium. (C) The Western blot analysis of VEGF/VEGFR1/VEGFR2 on corneal stroma. (D) Densitometry showing the fold increase of VEGF/VEGFR1/VEGFR2 expression levels compared to normal corneal stroma. No Tx 1 week and No Tx 2 weeks: No treatment group at 1 and 2 weeks after induction of corneal NV formation. Early Tx 1 week and Early Tx 2 weeks: In early treatment group, on week 1 and week 2 after bevacizumab injection. Late Tx 1 week and Late Tx 2 weeks: In late treatment group, on week 1 and week 2 after bevacizumab injection. Columns in C, D represent mean±standard error of the mean. *p<0.01

Western blot analysis showed the statistical results of VEGF/VEGFR1/VEGFR2 expression levels in the corneal epithelium and stroma compared to normal corneas in different experimental groups (Figure 5). The results is compatible with the immunohistochemical results, which demonstrated that the VEGF/VEGFR1/VEGFR2 expression in the corneal stroma is significantly higher than the normal cornea in the late treatment group (p<0.01). However, the expression remained as low as the normal corneal stroma in the early treatment group (p>0.05).

**Figure 6:**
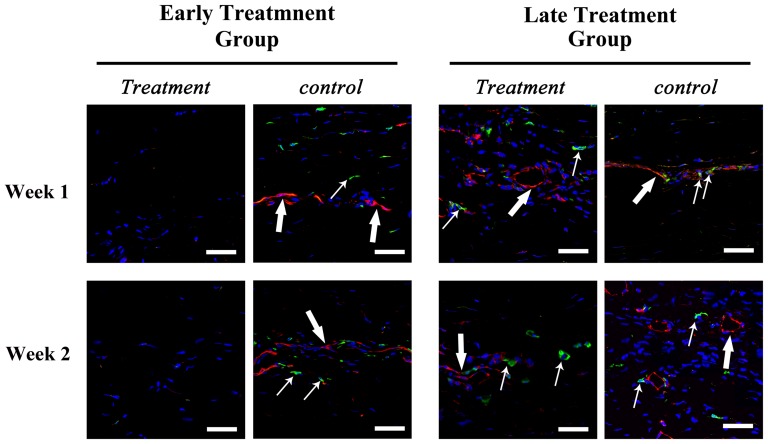
The effects of bevacizumab on macrophage infiltration. In early treatment group, bevacizumab significantly inhibited the infiltrate of AM-3K (+) cells, which represented macrophages. In late treatment group, there seemed to be no significant difference of macrophage infiltrate between treatment and control groups at week 1 and 2 after treatment. Thick white arrows: CD31 (+) vascular endothelial cells. Thin white arrows: AM-3K (+) macrophages. Red: CD31; Green: AM-3K; Blue: Hoechst33258 which stains nucleus. Scale bar: 40 μm. Three repeats were performed to ensure consistent results. In early and late treatment groups: Week 1, 2 = Week 1, 2 after bevacizumab injection.

In the early treatment group, bevacizumab significantly inhibited the infiltration of AM-3K-positive macrophages on week 1 and week 2 after treatment (p<0.01). In the late treatment group, bevacizumab appeared to have no effect on macrophage infiltration (Figure 6, Table 2).

**Table 2:** The effects of bevacizumab injections on the infiltration of AM3K (+) cells into the corneal stroma, and the expression of TUNEL (+) cells per field.

AM3K (+) cells/field						
	Early Treatment			Late Treatment		
	treatment	control	p-value	treatment	control	p-value
Week 1	0.6±0.3	12.5±1.3	<0.01	13.7±1.5	14.6±2.3	NS
Week 2	1.3±0.8	16.5±1.7	<0.01	12.0±1.3	14.3±3.6	NS

TUNEL (+) cells/field						
	Early Treatment			Late Treatment		
	treatment	control	p-value	treatment	control	p-value
Week 2	1.2±1.8	0.9±1.3	NS	1.7±2.5	3.8±3.1	NS
Week 4	0.7±0.9	1.6±3.1	NS	14.2±3.7	2.3±1.7	<0.01

AM3K (+) cells: macrophage. TUNEL (+) cells: apoptotic cells. NS: Statistically non-significant.

**Figure 7:**
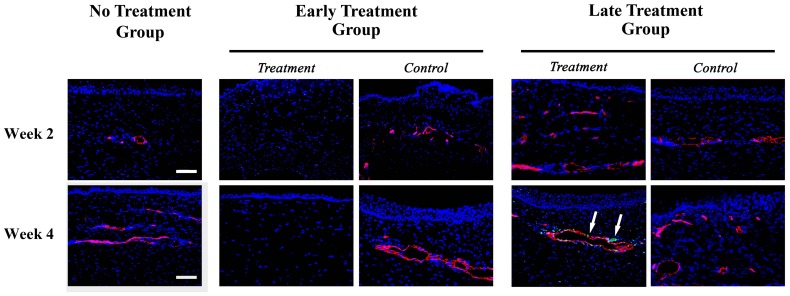
CD31 and TUNEL staining on cornea. In no treatment group, there was no staining of TUNEL on CD31 (+) vascular endothelial cells on week 2 and week 4. In early treatment group, bevacizumab significantly inhibited the growth of CD31 (+) vascular endothelial cells without the staining of TUNEL on week 2 and 4. In late treatment group, very few TUNEL (+) cells, indicating the apoptotic process, was found on CD31 (+) vascular endothelial cells week 4 after treatment (white arrows). However, there seems to be no TUNEL staining in the control group. Scale bar: 100 μm. Red: CD31; Green: TUNEL staining; Blue: Hoechst33258 which stains nucleus. Three repeats were performed to ensure consistent results. In early and late treatment groups: 2, 4 weeks = 2, 4 weeks after bevacizumab injection.

As for the possibility that bevacizumab may lead to vascular endothelial apoptosis, the co-staining of CD31 and TUNEL staining was found in vascular endothelial cells in the late treatment group at week 4 but not at week 2 after bevacizumab injection. There was almost no vascular endothelial apoptosis during the entire observational period in no treatment or early treatment groups. However, on week 4 in the late treatment group, there was significant difference of apoptotic cells between the treatment group and the control group (Figure 7, Table 2).

## Discussion

This study suggests that bevacizumab effectively inhibits corneal NV only if administered early, which is consistent with our previous report in the limbal insufficiency-induced corneal NV model. [27] Several mechanisms may explain this phenomenon. First, chronic NV may contain matured vessels covered with pericytes and smooth muscle cells. [45], [46], [59]–[62] The findings of our histochemical analysis showed that αSMA and HMW-MAA were expressed around the vascular endothelial cells as early as 2 weeks after the induction of corneal NV, which implies the early formation of matured vessels. In the absence of pericytes and smooth muscle cells coating, NV may regress when angiogenic factors are downregulated. [63]–[67] Hence, such coverage in corneal NV may decrease the anti-angiogenic effect of bevacizumab aimed at regressing newly formed immature vessels by blocking angiogenic factors.

Second, our immunohistochemical and Western blot results showed that VEGF, VEGFR1, and VEGFR2 were expressed in the epithelium of all corneas analyzed, regardless of the treatment condition (Figure 4, 5). Their expression co-localized with CD31-positive vascular endothelial cells on week 1 and week 2 after NV induction in the no treatment group and on week 1 and week 2 after bevacizumab injection in the late treatment group. Interestingly, there is limited VEGF, VEGFR1 and VEGFR2 expression in the corneal stroma in the normal cornea and the early treatment group, but relative strong expression in the no treatment and late treatment groups. Since bevacizumab almost completely inhibited the formation of corneal NV in the early treatment group, it is understandable that no vascular endothelial cells expressed VEGF, VEGFR1, or VEGFR2 under this circumstance. Our finding is different from those of very limited studies on the influence of bevacizumab on VEGF signaling. Kubota et al. reported that vascular endothelial cells lining along the fibrovascular membrane of proliferative diabetic retinopathy expressed VEGF, but the density of VEGF-positive cells decreased after intravitreal injection of bevacizumab. [44] Qian et al. reported that vitreous concentrations of VEGF decreased after intravitreal injection of bevacizumab in eyes with proliferative diabetic retinopathy. [42] The differences between the reported studies and ours could be explained by the strength and duration of NV induction. The influence of VEGF on maintaining corneal NV may be lesser during the chronic stage than during the acute stage, thus decreasing the therapeutic effects of the anti-VEGF antibody.

Third, the formation of pericytes or smooth muscle cells around the matured vessels found in chronic NV may affect the diffusion, persistence, and function of subconjunctivally injected bevacizumab. However, this explanation is refuted by our immunohistochemical result showing that the intracorneal distribution and persistence of bevacizumab was identical among normal corneas and among both early and late treatment groups.

Fourth, we found that bevacizumab could inhibit macrophage infiltration in early but not late treatment groups. Macrophages are known to trigger NV in inflamed or ischemic tissues including the cornea, [68]–[72] and bevacizumab has been reported to inhibit NV formation in inflamed or hypoxic corneal tissues. [22], [23], [26], [29] Since VEGF inhibitors are potent inhibitors of macrophage recruitment, [73]–[75] it is possible that the inhibition of macrophage infiltration plays an important role in the current study, especially in the early treatment group.

Fifth, several cytokines and mediators other than VEGF and different mechanisms may control the formation of corneal NV at different stages. [59], [76], [77] Anti-VEGF antibody alone may not be sufficient to inhibit corneal NV formation at various stages. [59], [76], [77] Finally, we did find that some vascular endothelial cells underwent apoptosis at 4 weeks after bevacizumab injection in the late treatment group (Figure 7), which implied that bevacizumab may still cause some vessels to undergo apoptosis and thus partially inhibit corneal vascularization in chronic NV. This finding is similar to that of Kohno et al., who showed that intravitreal injection of bevacizumab may induce endothelial apoptosis with vascular regression. [58] However, the extent of apoptosis of vascular endothelial cells might be too small to lead to clinically significant therapeutic effects. Interestingly, we did not find any apoptotic vascular endothelial cells at 2 and 4 weeks after bevacizumab injection in the early treatment group (Figure 7). This may lead to the conclusion that in the early treatment group, the inhibitory effects of bevacizumab on corneal vascularization are mainly caused by the “non-growth” of the corneal NV instead of “apoptosis” of the new growing vessels.

Our study has several limitations. First, the pathogenesis of corneal NV induced by closed eye CL wear may be different from other forms of corneal NV. Therefore, our study results may not be universally applicable to all types of corneal NV. Second, our animals had diffuse and extensive corneal NV, which may mask the trivial anti-angiogenic response of bevacizumab. Animal models with less extensive corneal NV may provide additional information. Third, we defined the criterion for chronic NV as a 1-month interval in this study. However, patients are frequently found to have a history of corneal NV for longer than 1 month. A longer interval between CL wear and initiation of treatment may be required to mimic clinical situations. Fourth, a high dose and frequent usage of bevacizumab was used to generate significant therapeutic effects in this study. Such an extensive treatment strategy may not be suitable for clinical use. Fifth, for experiment settings, more animal models, time points, and routes of bevacizumab application are needed to evaluate the therapeutic effect and underlying mechanism of bevacizumab-induced inhibition on the corneal NV formation. Our results demonstrated that early treatment with bevacizumab can prevent the formation of corneal NV, and late treatment can't inhibit the already formed corneal NV. Although our previous study confirmed that mid-term treatment with bevacizumab can inhibit new formed cornealNV at 1 week after stimulation in a limbal insufficiency mode [27], further studies are needed to apply the results to all clinical situations of corneal NV. Finally, subclinical inflammation in patients with corneal NV may be recurrent or persistent, which is different from our animal models. In spite of the above drawbacks, our study is valuable in several aspects. To the best of our knowledge, the finding that bevacizumab may affect the expression levels of VEGF/VEGFR1/VEGFR2 in corneal stroma differently in the early and late treatment conditions has not been reported before. We are also the first to demonstrate the effect of bevacizumab on macrophage infiltration and corneal endothelial apoptosis during corneal NV formation. All the findings point out the new understandings of the mechanism controlling the effects of bevacizumab on corneal NV formation.

In summary, our study showed the effects of bevacizumab in the treatment of extended closed eye CL-induced corneal NV. Early treatment may provide better therapeutic effect than late treatment. Diffusion of bevacizumab into the corneal stroma was found at least 1 week after injection, and decreased rapidly in both the early and late treatment groups without significant difference. Bevacizumab may inhibit macrophage infiltrate in the early treatment group, and caused limited vascular endothelial apoptosis in the late treatment group. On the basis of the results of our study, we suggest that pretreatment with bevacizumab at the early stage could inhibit corneal NV formation.
